# Editorial: Moral conflicts and ethical perspectives in dementia care

**DOI:** 10.3389/fpsyt.2025.1583226

**Published:** 2025-04-14

**Authors:** Sagit Lev, Milena von Kutzleben, Mark Schweda

**Affiliations:** ^1^ School of Social Work, Faculty of Social Sciences, Bar-Ilan University, Ramat Gan, Israel; ^2^ Division for Prevention and Rehabilitation Research, Department for Health Services Research, School VI - Medicine and Health Sciences, Carl von Ossietzky Universität Oldenburg, Oldenburg, Germany; ^3^ Division for Ethics in Medicine, Department for Health Services Research, School VI - Medicine and Health Sciences, Carl von Ossietzky Universität Oldenburg, Oldenburg, Germany

**Keywords:** dementia, ethical conflicts, autonomy, dignity, caregiving, advance directives (ads), assistive technologies

Dementia can be caused by a heterogeneous group of disorders and is characterized by a progressive deterioration of cognitive function and a concomitant loss of independence. Due to population aging, the syndrome is becoming more prevalent and receiving increasing attention in many contemporary aging societies. According to the World Health Organization (1), more than 55 million individuals worldwide are currently living with dementia, with nearly 10 million new cases diagnosed each year.

In addition to the clinical challenges associated with treatment, prevention, and care, dementia raises complex ethical concerns that warrant in-depth examination. The increasing cognitive impairments of those affected challenge common standards and procedures of autonomous decision-making and informed consent in medical ethics. The extensive and sophisticated requirements of good dementia care can put a strain on family carers as well as professional caregivers. Many societies are still pervaded by negative images of dementia that link the condition to social stigma and discrimination.

This Research Topic is dedicated to exploring these multifaceted ethical dimensions. The contributions tackle a variety of moral aspects and challenges of dementia care. They range from the significance of prominent ethical concepts like dignity, autonomy, or privacy in the context of dementia to moral conflicts arising in family or migrant live-in care arrangements to the use of new instruments, such as advance research directives or assistive technologies. The authors approach their topics from the perspectives of ethical analysis and empirical social research.

Buhr and Schweda explore the moral significance of privacy in the care of people with dementia. They argue that traditional concepts of privacy, which are primarily based on autonomy, may not be entirely appropriate in the ethical context of dementia care—particularly during the advanced stages of the disorder. Instead, they advocate for a more nuanced approach that considers remaining personal preferences, objective criteria of dignity and well-being, and the importance of maintaining meaningful relationships.

Barth’s ethnographic study explores the ethical dilemmas associated with managing challenging and aggressive behaviors of individuals with dementia. He critically examines the common practice of attributing such behaviors solely to pathological conditions rather than the patient’s free will. While this approach can protect patients by removing moral responsibility—thus preventing blame and preserving empathy and compassion—it may simultaneously compromise the dignity of individuals with dementia by denying their capacity for autonomous action and overlooking the underlying emotional and social needs. Barth proposes a balanced strategy that recognizes the disease’s influence on behavior while addressing the patient’s emotions, experiences, and desires.

Dogan et al. examine whether it is legitimate—or constitutes undue pressure—for an uninvolved daughter to assume caregiving responsibilities for her mother in situations where formal support is lacking. Their analysis interrogates the moral obligations that adult children may have toward their parents while also underscoring the ethical issues associated with involving previously uninvolved family members in caregiving. Moreover, they highlight the deeply political nature of this dilemma, noting that the scarcity of formal resources often forces professionals into making ethically problematic choices to alleviate the burden of dementia care.

von Kutzleben et al. investigate the dilemmas faced by migrant caregivers living in the homes of individuals with extensive support needs, often due to dementia. Their article offers a conceptual ethical framework for analyzing moral conflicts within the caregiver–patient–family triad. Specifically, the study discusses how tensions between the norms, values, and expectations of migrant caregivers, family members, and service recipients—operating across different social levels—can give rise to moral conflicts. This multidimensional approach facilitates a deeper understanding of the moral complexities involved in close care provided by migrant caregivers. It aims to inform policy improvements while offering targeted advice and support.

Furthermore, Ulitsa et al. explore the intricacies of triadic care arrangements involving dementia patients, foreign caregivers, and family members. By qualitatively analyzing interviews with 24 experts from Germany and Israel, the study examines six dimensions of vulnerability—namely, physical, psychological, relational/interpersonal, moral, socio-cultural-political-economic, and existential-spiritual. The findings indicate that all parties involved in care experience complex, interconnected vulnerabilities. Additionally, the study reveals similarities and differences in the experiences of experts from Germany and Israel, reflecting the influence of unique social and legal contexts on caregiving practices.

The third area of inquiry focuses on ethical issues related to advance directives. Vulliermet and Kenis offer a critical perspective on advance directives. They argue that discussions surrounding advance directives are sometimes framed in language that portrays dementia as “monstrous” or as an “enemy.” Such a portrayal not only perpetuates bias and stigmatization but also establishes a problematic dichotomy between the suffering of the “then self” and that of the “now self.” In response, they advocate for a more nuanced approach to advance directives that accounts for the needs and identity of the contemporary self of the individual with dementia.

Gieselmann et al. investigate the perceptions of individuals with mild cognitive impairment and their families regarding the benefits and challenges associated with advance research directives. Their findings indicate that participants recognize several advantages—most notably, the capacity of advance research directives to alleviate the decision-making burden on family members and uphold personal autonomy. However, the study also reveals significant challenges, including the potential for conflicts between current preferences and the instructions documented in these directives.

Finally, two articles address the ethical dilemmas associated with the use of assistive technologies and robotics in dementia care. Deusdad’s review examines the integration of technologies—including social and companion robots—into dementia care. It addresses the technical, psychological, and sociocultural dimensions of human-robot interaction among older adults with dementia, highlighting ethical concerns regarding robots’ capacity to interpret human needs, issues of informed consent, increased dependency, and difficulties distinguishing reality from simulation. The review also discusses the potential ethical impact of reducing human caregivers’ roles.

Welsch and Schicktanz conducted interviews with experts to examine the conditions that both promote and hinder the deployment of intelligent assistive technologies in this context. Their findings reveal a complex interplay of accelerating and inhibiting factors operating at three distinct levels: societal, political-regulatory-economic, and technological. These results underscore the need to enhance facilitators and mitigate barriers across all three domains.
